# Parenting Acceptance and Commitment Therapy Online (PACT Online) for parents of children diagnosed with or with increased likelihood of neurodevelopmental disability: study protocol of a randomised controlled trial

**DOI:** 10.1136/bmjopen-2024-088981

**Published:** 2025-06-20

**Authors:** Koa Whittingham, Grace Kirby, Roslyn N Boyd, Iona Novak, Amy E. Mitchell, Natasha Reid, Syed Afroz Keramat, Kristelle Hudry, Josephine Barbaro, Jacqui Barfoot, Robert S Ware, Fiona Russo, Helen Heussler, Andrea McGlade, Ashleigh Bullot, Megan MacDonald, Tommy Tran, Sophie Harrington, Jeanie Sheffield, Rebecca Olson, Nathalia Costa

**Affiliations:** 1 Queensland Cerebral Palsy and Rehabilitation Research Centre, Child Health Research Centre, Faculty of Medicine, The University of Queensland, Brisbane, Queensland, Australia; 2 Cerebral Palsy Alliance, The University of Sydney, Sydney, New South Wales, Australia; 3 School of Nursing, Midwifery and Social Work, University of Queensland, Brisbane, Queensland, Australia; 4 Child Health Research Centre, Faculty of Medicine, The University of Queensland, Brisbane, Queensland, Australia; 5 Centre for Health Services Research, Faculty of Medicine, The University of Queensland, Brisbane, Queensland, Australia; 6 Department of Psychology, Counselling and Therapy, School of Psychology and Public Health, La Trobe University, Melbourne, Victoria, Australia; 7 Olga Tennison Autism Research Centre, School of Psychology and Public Health, La Trobe University, Melbourne, Victoria, Australia; 8 Griffith Biostatistics Unit, Griffith University, Brisbane, Queensland, Australia; 9 School of Business, University of Southern Queensland, Toowoomba, Queensland, Australia; 10 Paeds in a Pod, Brisbane, Queensland, Australia; 11 AEIOU Foundation for Children with Autism, Brisbane, Queensland, Australia; 12 BUSHKids, Brisbane, Queensland, Australia; 13 NOFASD Australia, Brisbane, Queensland, Australia; 14 School of Psychology, University of Queensland, Brisbane, Queensland, Australia; 15 School of Social Science, The University of Queensland, Brisbane, Queensland, Australia; 16 University of Queensland’s Clinical Trial Capability Team (ULTRA Team), The University of Queensland, Brisbane, Queensland, Australia

**Keywords:** Developmental neurology & neurodisability, Parents, Psychological Stress

## Abstract

**Introduction:**

Approximately 1 in 13 Australian children have a neurodevelopmental disability. This project aims to assess the effectiveness and implementation of an online parenting support programme, Parenting Acceptance and Commitment Therapy (*PACT*) *Online,* for parents of children with neurodevelopmental disabilities for improving the parent–child relationship and parent and child outcomes.

**Methods and analysis:**

This hybrid type 1 randomised controlled trial will focus on evaluating intervention effectiveness and understanding the context for implementation. The primary outcome is observed emotional availability within parent–child interactions assessed at postintervention (12 weeks postbaseline) with additional measurement at follow-up (6 months postbaseline). Secondary outcomes include (1) parent-reported emotional availability, (2) parental mindfulness, (3) parent mental health, (4) psychological flexibility, (5) adjustment to child’s disability, (6) health behaviour and (7) regulatory abilities as well as child outcomes of (1) mental health, (2) adaptive behaviour and (3) regulatory abilities. Evaluation of implementation will include an economic evaluation of costs and consequences, and an implementation analysis grounded in the consolidated framework for implementation research with a focus on contextual factors influencing implementation.

**Ethics and dissemination:**

Ethical approval has been obtained from the University of Queensland Human Research Ethics Committee (023/HE000040). Dissemination of study outcomes will occur through the appropriate scientific channels. Long-term implementation will be grounded within the implementation analysis and occur in partnership with the partner organisations and consumer engagement panel. This will include releasing the *PACT Online* intervention as a massive open online course on the edX platform if support for intervention effectiveness and implementation is found.

**Trial registration number:**

ACTRN12623000612617; this trial has been registered with the Australian New Zealand Clinical Trials Registry.

**STRENGTHS AND LIMITATIONS OF THIS STUDY**

- This study is an appropriately powered randomised controlled trial of an online intervention that has already been successfully piloted.
- Outcomes include a parent–child interaction observation coded by accredited coders blinded to condition, parent report measures and parent and child heart rate variability as a physiological marker of self-regulation.
- Adoption of a waitlist control design (for ethical reasons) means that it will not be possible to conduct further follow-up of research participants limiting potential for conclusions to be drawn beyond the 6-month follow-up period outlined in this protocol.
- This trial includes an implementation analysis with the aim of identifying relevant contextual factors influencing implementation and implementation strategies.

## Introduction

 Approximately 1 in 13 Australian children^1^ have a neurodevelopmental disability (NDD) including cerebral palsy (CP), autism, fetal alcohol spectrum disorder (FASD), attention-deficit hyperactivity disorder (ADHD) and intellectual disability. NDDs are lifelong and require ongoing support at significant cost (approximately $A35 000 per child per annum).^2^ Children with NDDs have a four to fivefold increased risk of poor mental health including behavioural problems^3^ and a 2-fold to 20-fold increased risk of poor physical health.^4^ In addition, they are at increased risk for adverse life outcomes in education and employment.^5^

### The family context of NDDs

There is a wealth of research demonstrating that parenting is important and that supporting parents improves child development and mental health.^6^ For children with NDDs, parents are essential partners in the delivery of health and educational supports, services and interventions.^7^ That is, effective support for children relies on parental implementation and ‘good parenting’. Parents seek supports, manage their child’s interdisciplinary health and educational teams, advocate for their child at school and in community contexts and implement programmes and strategies in the home. More recently, in Australia, parents select supports and services for their child on the National Disability Insurance Scheme (NDIS).^8^ Supports for the child may bottle-neck if there are limitations in parent capacity for implementation or challenges within the parent–child relationship.^9^

As is true of all parents, the ability of parents of children with NDDs to parent well is underpinned by their own physical and mental health.^10^ Parents of children with NDD are at a four to fivefold increased risk of compromised physical and mental health^11^ and many experience a grief that may compromise their own well-being and parenting.^12^ Ongoing psychological adaptation and complexity, including increased demands on the parent, discrimination or social isolation, can increase over time and can lead to a lifestyle that is detrimental to the health and well-being of parents,^13^ with flow-on effects for parenting and the parent–child relationship.

NDDs also impact on the emotional availability of the parent–child relationship; that is, the emotional signalling within the relationship, through the child.^14^ Children with NDDs can be slower to respond to parental cues, can cue in an atypical way or be less skilled at eliciting their parent’s involvement.^15^ Understandably, parents may respond by becoming more directive and intrusive, a phenomenon that is not in the child’s best interests as it does not encourage growth of the child’s initiative. Thus, the parent–child relationships of families of children with NDD require support, but support that is appropriate to their specific challenges.

Despite this, parenting support is not offered as standard for families of children with NDDs, and in Australia, the NDIS does not fund parenting interventions.^8^ Parents of children with NDDs are forced to seek mental health support through the general health system, a process that many report does not meet their needs.^16^ Parents of children with NDDs want specialist support for their mental health, well-being and parenting concerns: support that is focused on the specific challenges of parenting a child with an NDD and is accessible and flexible in delivery.^16^

### Tailor-made parenting support grounded in acceptance and commitment therapy

Parenting support is important for families of children with NDDs and parenting interventions are effective for families of children with NDDs.^17^,^19^ Specifically, parenting interventions incorporating Acceptance and Commitment Therapy (ACT) have been found to be effective for families of children with NDDs including acquired brain injury,^17 20^ autism^21 22^ and CP,^19 23^ both offered as standalone^21 22 24^ and in combination with behavioural parenting interventions.^19 23^ ACT is a form of cognitive behavioural therapy focused on enhancing an individual’s psychological flexibility^25^ or the ability to persist with or change behaviour, with full awareness of the present moment and in the service of chosen values.^26^ Core to ACT is acknowledgement of the universality of suffering, and the importance of living a meaningful life through effective action and focusing on what works, themes that resonate for many parents of children with NDDs.

This paper reports the protocol for a type-1 hybrid randomised controlled trial (RCT) of *Parenting Acceptance and Commitment Therapy (PACT) Online*: tailor-made ACT-based parenting support developed specifically for parents of children with NDDs that has been built for ease of implementation in existing health and disability systems. We trialled an early version of *PACT Online* with 68 families of children aged 2–12 years with CP, demonstrating an intervention effect of improved emotional availability in the parent–child relationship; specifically, reductions in parental intrusiveness and improvements in child involvement^24^ exactly the aspects of emotional availability at risk in NDD.^15^ In addition, we also found improvements in parental mindfulness and parent-reported child quality of life in the ‘well-being and acceptance’ and ‘participation and physical health’ domains.

### Aim

This hybrid type 1 RCT will test the effectiveness and implementation of *PACT Online* with families of children aged 0–10 years diagnosed with, or with an increased likelihood of, an NDD, compared with a waitlist control (care as usual; CAU) group.

### Hypotheses

We hypothesise that families allocated to the *PACT Online* group, compared with families in the CAU group, will show improved emotional availability on the observational measure of the Emotional Availability Scale (EAS;^15^ immediately postintervention (T2; 12 weeks postbaseline, primary outcome). Furthermore, we predict that this effect will be sustained at follow-up (T3; 6 months post-baseline).

We hypothesise that families in the *PACT Online* group, in comparison to CAU, will show improved parent-reported emotional availability on the Emotional Availability–Self-Report (EA-SR;^27^ as well as improved parent outcomes across measures of (1) parental mindfulness, (2) parent mental health, (3) psychological flexibility (4) adjustment to child’s disability, (5) health behaviour and (6) regulatory abilities and improved child outcomes across measures of (1) mental health, (2) adaptive behaviour and (3) regulatory abilities at T2 (12-week postbaseline) and T3 (6-month postbaseline).

To describe and understand the context for implementation, we will consider the costs and consequences as well as the contextual factors that may influence implementation. Analysis of contextual factors will be guided by the consolidated framework for implementation research (CFIR)^28^ and will consider feasibility, acceptability and appropriateness of PACT Online, sustainability potential and contextual factors that could impact on implementation success.

## Methods and analysis

### Study design

**Figure 1:**
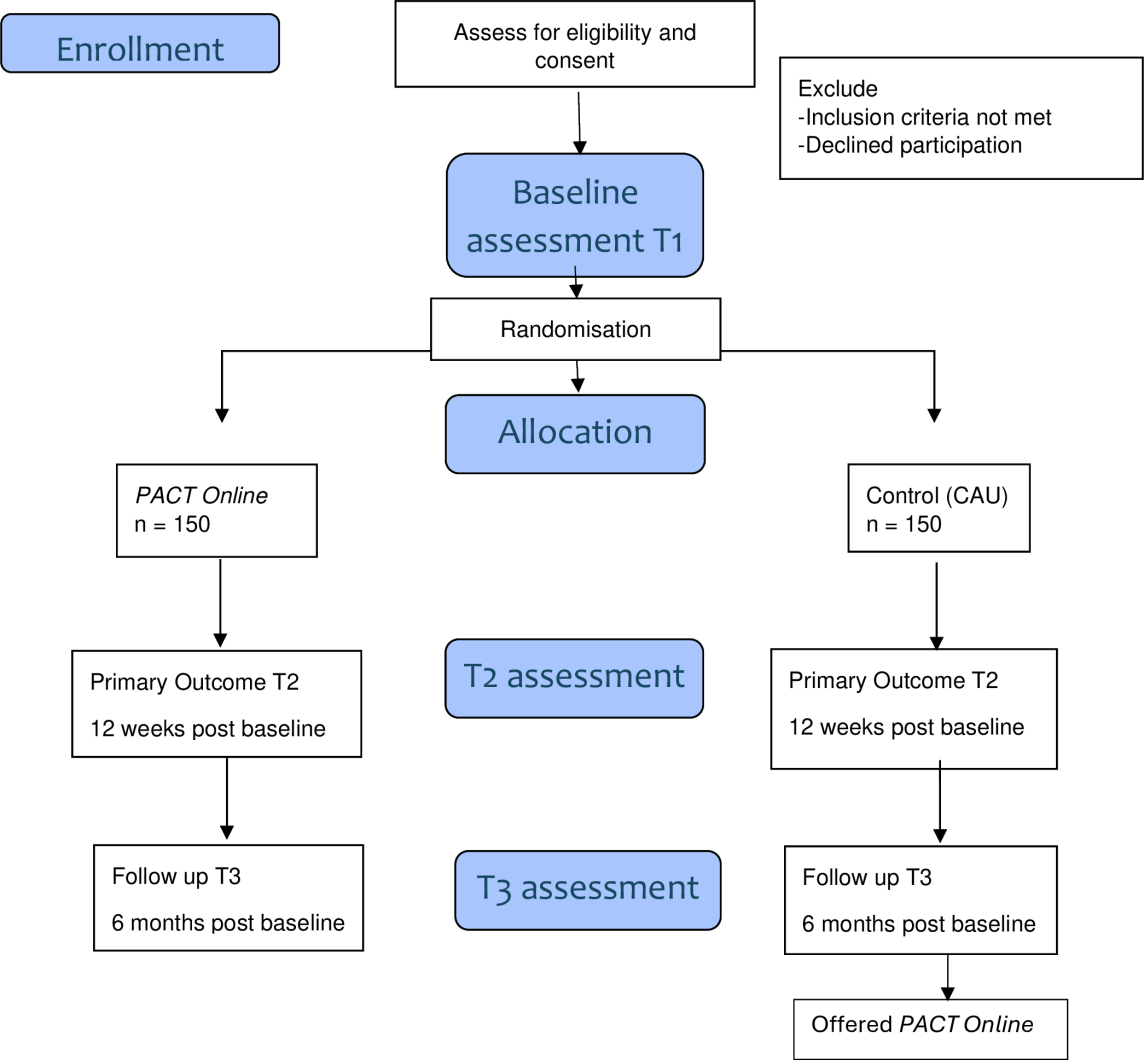
Consolidated Standards of Reporting Trials (CONSORT) flowchart for PACT Online study. CAU, care as usual; PACT, Parenting Acceptance and Commitment Therapy.

This study is a hybrid type 1 RCT. A type 1 hybrid is focused on effectiveness while also exploring implementation. We will conduct an RCT of *PACT Online* with 300 families of children with NDD. This is a two-arm RCT in which families of a child (0–10 years) with a diagnosis of an NDD or assessed as having an increased likelihood of an NDD will be randomly assigned to *PACT Online* or CAU, with follow-up until 6 months postbaseline. All families will complete assessment at preintervention baseline (T1), immediate postintervention outcome (T2; 12 weeks postbaseline for CAU group) and follow-up (T3; 6 months postbaseline). As the *PACT Online* programme is secure and unable to be accessed by external parties during the study period, no between-group contamination is possible. Consolidated Standards of Reporting Trials guidelines will inform the conduct and reporting of this RCT (see figure 1).

### Recruitment

Recruitment will be conducted with cooperation from partner organisations across multiple sectors including primary care (Paeds in a Pod), community-based allied health (BUSHKids; AEIOU Foundation for Children with Autism) and parent support organisations (NOFASD Australia, CP Alliance) as well as through broad recruitment strategies including National Registers such as the Australian CP Register, the Aus-CP-CTN Centre of Research Excellence, schools and a media campaign. While researchers are situated at key centres in Brisbane, Sydney and Melbourne, due to the fully online nature of the intervention and the assessment protocols, recruitment will be possible Australia-wide. Australia-wide recruitment, recruitment through schools and deliberate recruitment from regional and rural areas via BUSHKids will maximise representativeness of the sample. It should be noted that generalisability will be limited to English-speaking families. For each family, there must be at least one parent participant where *parent* is defined by the performance of a parental role. Hence, in this study, *parent* is inclusive of custodial grandparents or stepparents. Single parent families will be eligible, where families have more than one parent or another significant familial caregiver such as a non-custodial grandparent, a secondary participant may be nominated. For families with a primary caregiver, the participation of the primary caregiver will be strongly encouraged, and they will be treated as the primary participant for assessment purposes. Informed consent will be obtained from all participating parents prior to commencement in accordance with institutional ethics procedures (see online supplemental file for a copy of the participant consent form). Recruitment began on 26 February 2024 and it is planned to continue until approximately mid-2026.

### Inclusion criteria

To participate, families must have a child 0–10 years of age with either a diagnosis of any NDD (eg, CP, Autism, FASD, ADHD) or at identified increased likelihood of an NDD on the basis of one of the following: (1) a diagnosis of developmental delay as defined by Australian NDIS (ie, substantial reduction in functional capacity) in two or more domains (ie, gross motor, fine motor, language, or personal/social); (2) an increased likelihood of CP as established through the General Movements Assessment^29^ or Hammersmith Infant Neurological Examination;^30^ (3) an increased likelihood of autism as established through the Social Attention and Communication Surveillance Revised,^31^ including the ASDetect mobile application^32^ or (4) identified as being at risk of FASD in accordance with the Australian Guide to Diagnosis of FASD.^33^ These inclusions are consistent with the latest practice and international Clinical Practice Guidelines for the early identification of CP,^34^ autism^35^ and FASD.^33^ The age range of 0–10 years was chosen, so that PACT Online could be available as early as possible, as soon as NDD was suspected to developmental delay identified.

### Exclusion criteria

The exclusion criteria have been deliberately limited to only those necessary to ensure that the sample is appropriate to answer the research question. Parents who are unable to understand the written information sheet will necessarily be ineligible for the study, as the intervention includes materials written in English and study participation also requires the completion of sets of online questionnaires written in English. Any families ineligible for the study based on language will be referred to other services, for example, parenting programmes or allied health services, as appropriate. The *PACT Online* intervention and study assessment protocols will all be conducted online. Hence, families who do not have sufficient internet access to participate in the study, either in their own homes or elsewhere, will also necessarily be ineligible and referred to other services as appropriate. No other exclusion criteria are imposed.

### Sample size

The sample size calculation is based on the between-group difference for the primary outcome, the global relationship quality score from the EAS, which ranges from 0 (worst relationship possible) to 100 (best relationship possible). Based on a previous RCT conducted by our team in 67 families of children with an NDD (CP),^24^ we assume the SD of the Global EAS will be 14.3 units, and that identifying a difference between the active (PACT) and waitlist control group of 5.0 units is clinically important. This is equivalent to a between-group difference of 0.35 SD. To achieve 80% power, 258 families (129 in each group) are required to provide outcome data (alpha=0.05). To allow for expected 11%–15% participant attrition (as experienced in our previous trials),^24^ we will recruit a total of 300 families into this RCT. Participant retention strategies will be guided by the consumer engagement panel and will include a quarterly study update. Sample size calculations were conducted using Stata statistical software V.14 (StataCorp, College Station, Texas).

### Randomisation and blinding

Following screening to ensure eligibility, consent and the completion of baseline assessment, families will be randomised to *intervention* or CAU using central computer-generated block randomisation (Griffith University). Allocation will occur in a 1:1 ratio and blocks will be of size 12 or 16, with size randomly selected. Neither participants nor clinicians can be blind to the group allocation, and we acknowledge this may be a confound. Scoring on the observational EAS (primary outcome) will be conducted by coders who will be blind to participant group allocation.

### Intervention: *PACT Online*

*PACT Online* is an online/telehealth parent support intervention developed in partnership with parents and focused on empowering parents as well as supporting parenting, parental mental health and the parent–child relationship. It is theoretically grounded in ACT and was developed by authors Dr Koa Whittingham and Dr Jeanie Sheffield. PACT Online as tested in this RCT is an enhanced and adapted intervention of PACT as tested in an RCT with 68 families of children aged 2–12 years with CP.^24^

*PACT Online* is delivered over 12 weeks via the edX platform (www.edx.org/) and six telehealth sessions. It includes core ACT content and adopts a flexible, goal-directed approach focusing on each parent’s tailored behavioural goals. Parents complete six modules over 12 weeks, a module per fortnight, with an individual telehealth session for each module. In the individual telehealth sessions trained allied health clinician provide individualised feedback and additional support, helping families to apply the content to their lives. *PACT Online* thus offers a translatable and scalable universal online support at little to no cost, with additional clinician-delivered telehealth support for families who need it. For more details, see table 1.

**Table 1:** PACT Online: core content and delivery

Content	Delivery
Parents will be supported to grow *parent capacity* by cultivating *flexible, sensitive and effective parenting* through developing: Effective strategies for teaching new skills and encouraging positive behaviour Effective strategies for managing misbehaviour Effective strategies for interacting with the wider community Awareness of their own and their child’s (developing) values Awareness of effective action, ie, what *works* Insightfulness, understanding the child’s perspective Experiential acceptance, the ability to live with distressing emotions, cognitions and memories as they arise, and continue to focus on effective action Acceptance of their child and their child’s emotions, cognitions, etc. Self-compassion and achievable self-care repertoires	*PACT Online* EdX course**:** 12-week EdX course, includes videos, text, multiple choice questions, real-life activities (eg, practising mindfulness during a parent–child interaction), and a discussion board facilitated by the *PACT Online* clinicians
	Clinician support**:** Clinician consultations (30–60 min) will support parents in understanding and acting on *PACT Online* content, taking a flexible goal-directed approach.Clinician consultations will focus on parental goals, parental application in real life, overcoming obstacles and integrating flexibility into parent–child interactions. Clinician consultations will be conducted fortnightly via ZOOM. They will be recorded and assessed for adherence to protocol.
Parents will choose *three goals for change***:** (i) a parent–child relationship or parenting goal, (ii) a parental self-care or health behaviour goal and (iii) a child behaviour/adjustment goal.	

PACT, Parenting Acceptance and Commitment Therapy.

### Fidelity

The *PACT Online* edX course is static and hence remains the same for each participant who accesses it. For the telehealth consultations, all therapists will follow a consultation manual and will obtain clinical supervision from Dr Koa Whittingham, Dr Jeanie Sheffield and Dr Jacqui Barfoot to ensure fidelity. Dr Jacqui Barfoot will also be the therapist for some families in the study. Records of intervention delivery and online course access will be kept, and telehealth sessions will be recorded.

### Control group: CAU

Families randomised to the CAU (waitlist control) group will receive their usual services (eg, medical, allied health, therapy) during the study participation period and will be offered the *PACT Online* intervention after completing the T3 follow-up assessment (at 6 months postbaseline). We will collect information on which services each family accessed throughout the study.

### Adverse events

Any adverse events will be recorded and reported in accordance with ethical procedures. The trial will be audited every 6 months from the beginning of recruitment by a clinical trials monitor indepedent of the study team.

### Patient and public involvement

*PACT Online* was developed grounded in parent feedback collected through our qualitative research,^16^ during which parents told us they wanted a parenting support intervention that was flexible and specific to their needs as parents of children with NDD. The intervention was refined grounded in further qualitative feedback from participants in the first PACT trial.^24^ From the outset, the current project has included a consumer representative (FR), who has also established a consumer engagement panel, which will be maintained throughout the project. The role of the consumer engagement panel involves providing feedback on intervention materials, assessments and assessment burden, and all participant-facing study documents (eg, information and consent forms, recruitment materials). In addition, the consumer engagement panel will be consulted on public dissemination of the study results, including the implementation recommendations.

This project also includes partner organisations including primary care (Paeds in a Pod), community-based allied health (BUSHKids; AEIOU Foundation for Children with Autism) and parent support organisations (NOFASD Australia, CP Alliance). All partner organisations have been involved in the study from the outset and have/will input into study design and documents, recruitment, dissemination of study results and the translation of outcomes into clinical practice. All partner organisations will be offered access to and training in *PACT Online* at the conclusion of the study. This training will be grounded in the implementation analysis.

### Outcome measurement

All assessments will be conducted remotely and/or online using REDCap for questionnaires and Zoom videoconferencing for collection of the EAS observational assessment footage and will be stored on REDCap and/or University of Queensland (UQ) servers with state-of-the-art data security meeting the requirements of the National Health and Medical Research Council and the Australian Code for Responsive Conduct of Research and accessible to study personnel only. Data will be destroyed after 15 years in accordance with UQ policy. In families with two participating parents, families will be encouraged to nominate the primary caregiver as the primary participating parent where applicable. The primary participating parent will complete all assessments at all time points. The secondary participating parent will complete a shorter online questionnaire (including measures of emotional availability, parental mindfulness, parent mental health, psychological flexibility, adjustment to child’s disability and health behaviour as well as economic evaluation measures) to minimise family burden. The questionnaires take approximately 1 hour to complete in total. The limitations of self-report measures are acknowledged.

### Baseline characteristics

*Demographic information* including family characteristics (eg, parent level of education, number of children), child diagnoses and perinatal risk factors (eg, alcohol consumption assessed using the AUDIT-C, pregnancy/birth complications) will be collected via questionnaire.

*The Gross Motor Function Classification System* is a parent-rated measure using a five-level system to classify children by age-specific gross motor ability, with published evidence of reliability and validity.^36^ It will be used to classify the functional abilities of all children with a physical disability.

### Primary outcome

#### Emotional availability

*EAS* measures the quality of the parent–child relationship across six scales: parental sensitivity, parental structuring, parental non-intrusiveness, parental non-hostility, child responsiveness and child involvement^15^ and a global relationship quality score. A 20 min naturalistic observation of parent–child interaction in the home will be recorded via Zoom and subsequently coded by experienced and accredited EAS coders, kept blind to the intervention condition and timepoint. Parents will be advised that during the recording, they should interact with their child in an ordinary, everyday way. For example, they could choose to play with toys, read a book, draw or perform any other interactive activity. Any child behaviour should be dealt with as they normally would. Extensive use of the EAS has demonstrated the excellent construct validity of this scale in establishing the expected relationship between EA and parent–child attachment, including in naturalistic observations in home settings.^15 37^ The EAS is appropriate for the full range of child ages in this study (0–10 years) with specific manuals guiding how to apply the same constructs for early (0–5 years) and middle (6–17 years) childhood. Our research group has achieved fair to excellent inter-rater reliability access the six scales in a similar study: parental sensitivity (ICC=0.72), parental structuring (ICC=0.51), parental non-intrusiveness (ICC=0.69), parental non-hostility (ICC=0.82), child responsiveness (ICC=0.82) and child involvement (ICC=0.71).

### Secondary outcomes

*EA-SR* (*36 items*) uses parent-report to measure emotional availability within the parent–child relationship.^27^ The EA-SR produces five subscales: mutual attunement, affect quality, capacity to involve the parent, intrusiveness and hostility. Responses are given on a 5-point scale ranging from 0=*not at all agree* to 4=*totally agree,* with a mid-point of 2 being *Neutral*. Good reliability estimates for the subscales have been found ranging from 0.71 to 0.84, with the exception of the affect quality subscale (α=0.49),^27^ which will be excluded from analysis if sufficient reliability is not achieved. The EA-SR has excellent validity and has been validated against the EAS.

#### Parental mindfulness

*The 10-item Interpersonal Mindfulness in Parenting (IM-P*) *Scale* assesses a parent’s ability to maintain present-centred attention and emotional awareness during parent–child interactions.^38^ The IM-P produces four subscales: present-centred attention, present-centred emotional awareness, non-reactivity/low-reactivity and non-judgmental acceptance. The IM-P has acceptable reliability (α=0.72) and strong support for its concurrent and discriminant validity has been obtained.^38^

#### Parent mental health

*The Depression Anxiety Stress Scales (DASS-21; 21 items*) assess symptoms of depression, anxiety and stress in adults.^39^ It comprises three scales, each with good internal consistency: the depression (α=0.91), anxiety (α=0.84) and stress (α=0.90) scales, and has good discriminant and concurrent validity when assessed against other validated depression and anxiety measures.^40^

#### Parent psychological flexibility

*Comprehensive Assessment of Acceptance and Commitment Therapy Processes (Com-PACT; 23 items*) measures psychological flexibility as core processes: openness to experience, behavioural awareness and valued action.^41^ It has good internal consistency (α=0.87–0.90) and good construct and convergent validity against psychological flexibility and distress measures (eg, a correlation of *r*=0.65 was obtained between the Com-PACT and the depression subscale of the DASS-21).^41^

#### Parent adjustment to child’s disability

*Parent Experience of Child Illness Scale (25 items*) assesses parental adjustment to a child’s chronic illness. An adapted disability-specific version will be used.^42^ It produces four domain scores: emotional resources, long-term uncertainty, guilt and worry, and unresolved anger and sorrow. It has acceptable reliability (α=0.72–0.89) and construct validity.

#### Parent health behaviour

*Good Health Practices Scale (16 items*) measures health behaviour in adults and has been validated in community samples, including by correlating with physiological biomarkers of poor health.^43^ It is summed to create a total score of 80, with higher scores indicating better health practices.

#### Parents’ goals for change

*Goal Attainment Scale (GAS)* will be used to identify three goals for change: (1) parent–child relationship or parenting goal, (2) parental self-care or health behaviour goal and (3) child behaviour/adjustment goal. Parents will use structured monitoring sheets to track frequency and/or duration of target behaviours, with final scores ranging from 0% (no improvement) to 100% (total success/goal attained). GAS has convergent validity against observational and parent-report measures and can be used to assess change across different types of behaviours.

#### Parent and child regulatory abilities

*Heart rate variability (HRV)*: changes in parent and child self-regulatory abilities will be assessed via HRV, an important physiological index of self-regulation,^44^ with higher HRV (ie, greater variability of the inter-beat intervals) related to better cognitive and mental health outcomes. HRV data will be collected using the Polar H10.

#### Child mental health

*Child Behaviour Checklist (CBCL parent report; 113 items*) assesses child behavioural and emotional problems: affective problems, attention deficit/hyperactivity, anxiety, oppositional defiance, somatic problems and conduct problems.^45^ The CBCL is well validated and appropriate for younger (1.5–5 years old) and older (6–18 years old) children, with age-specific versions.^45^

#### Child adaptive behaviour

*Vineland Adaptive Behaviour Scale Comprehensive (third edition VABS-III parent report*) is a measure of adaptive behaviour from birth to adulthood.^46^ Standard scores range from 20 to 160 for the four domains of communication, daily living skills, socialisation and motor skills, and an overall Adaptive Behaviour Composite. It has good internal consistency, test–retest reliability, inter-interviewer reliability and validity.^46^

### Implementation factors

#### Health economic evaluation

***Caregiver burden.** Carer Experience Scale (CES*) will be employed as a measure of caregiver burden. This validated measure of care-related quality of life has six domains (activities, support, assistance, fulfilment, control and relationship with the care recipient).^47^ The CES is scored from an algorithm based on norms in the general population and will be used to assess carer outcomes in economic evaluation.

**Health services utilisation**. The *Health Resource Use* questionnaire has been custom-built for the current research and will be used to collect healthcare utilisation information starting from baseline and for the duration of the study follow-up period. This questionnaire includes questions relating to the use of allied health services for children (eg, frequency, duration and cost of health services such as physiotherapy and paediatricians), mental health support for parents (eg, use and costs associated with mental health services such as psychologists and general practitioners) and parents’ satisfaction with the healthcare services they are currently accessing for both themselves and their child.

**Productivity loss**. Health and Work Performance Questionnaire (HPQ) will be used to measure parents’ productivity loss,^48^ a time cost calculated through absenteeism (ie, parent missed time from work) and presenteeism (ie, parent at work but less productive than usual and compared with others). The WHO HPQ has been widely used to assess absenteeism and presenteeism behaviours of workers. The HPQ has been found to have good reliability and validity as well as being sufficiently sensitive to change over time.^49^

**Health-related quality of life**. Parent health-related quality of life will be measured through the EuroQol five-dimension five-levels (EQ-5D-5L) questionnaire.^50^ The EQ-5D-5L questionnaire is a widely used preference-based generic scale that describes and assesses an individual’s health state across five dimensions: mobility, self-care, usual activities, pain/discomfort and anxiety/depression. Each is assessed through a five-level scale (no, slight, moderate, severe or extreme problems), with the uppermost level, indicating the greatest level of severity. Responses to the questionnaire are used to form a single utility score (EQ-5D-5L tariff) ranging from negative (less than zero) to 1.0 (full health) as an indicator of health status. A higher EQ-5D-5L utility score implies better health status.

#### Acceptability, appropriateness and feasibility

Questions assessing acceptability, appropriateness and feasibility are built into the *PACT Online* edX platform. Participants will be asked to rate each module in terms of usefulness on a 10-point Likert scale. In addition, they will have the opportunity to give written feedback in terms of usefulness, ideas for improvement and inclusivity.

#### Qualitative analysis of contextual factors for implementation

A purposive sample of parents in the *PACT Online* group will participate in semistructured interviews with two independent researchers (RO and NC) not involved in project management or intervention delivery. To gain in-depth insight into factors shaping implementation acceptability, interviews were selected to support a CFIR qualitative study design,^28^ prioritising individuals’ experiences of the intervention, settings and processes. Purposive sampling criteria (eg, age of child, carer relationship) will be defined iteratively after the first 10 interviews. Based on the ‘information power’ required,^51^ it is anticipated that recruitment will conclude after 30–40 inerviews. Grounded in CFIR,^28^ the interview guide prioritises discussion of acceptability, appropriateness and feasibility of implementing the intervention with parents as well as identifying contextual factors that act as facilitators and barriers to implementation success within the individual, inner setting and outer setting domains (see online supplemental materials for interview guide). The same independent researchers will also conduct qualitative interviews with the therapists delivering PACT Online and with some stakeholders from partner organisations as well as review a randomly selected subset of clinician notes.

### Statistical analysis of primary and secondary outcomes

The primary outcome will be analysed using mixed-model repeated-measures linear regression (multilevel modelling) with time (T1, T2, T3) and group (*PACT Online*/CAU) included as the main effects, and a time-by-group interaction term. Child will be included as a random effect to account for the repeated measures from each participant. Secondary comparisons will be conducted using linear models for continuous outcomes, logistic regression models for binary outcomes and Poisson models for count outcomes. The value of the outcome variable at baseline will be included as a covariate when appropriate. When repeated measures are analysed, the non-independence of results from the same family will be accounted for using mixed-effects models. Subgroup analyses will compare outcomes by child age and child diagnosis. Analysis will follow standard principles for RCTs with two-group comparisons using the intention-to-treat principle. In the event that >10% of outcomes are missing, we will conduct a sensitivity analysis to investigate the sensitivity of results. The pattern of missingness will be examined, and if the data are ‘missing at random’, we will use multiple imputation by chained equations, whereas if the data are determined to be ‘missing not at random’, we will use pattern mixture models.

#### Health economics evaluation

The primary purpose of economic evaluation will be to conduct a cost and consequence analysis (CCA) to guide the allocation of resources for implementation, with CCA the chosen approach because the intervention has multiple health and non-health outcomes that are hard to measure in a single unit. CCA will assess a wide range of important health outcomes to assess the value for money of the intervention. The study will look at the incremental differences in various health outcomes between the intervention and CAU and present a comprehensive summary.

#### Implementation analysis

Qualitative analyses will be conducted on (1) semistructured interviews with a subgroup of *PACT Online* participants and (2) semistructured interviews with therapists delivering PACT Online, (3) semistructured interviews with stakeholders in partner organisations and (4) a randomly selected subset of clinician notes. The CFIR, a meta-theoretical framework synthesising implementation theories, will be used to inform coding and analysis with a focus on ensuring that (1) *PACT Online* is acceptable and appropriate for parents, meeting their needs and (2) the contextual factors shaping implementation in existing health, educational and disability services are understood, with appropriate implementation strategies developed. For example, parents will be asked what the best, worst and most difficult parts of PACT Online were, what changes they have noticed, whether or not they found PACT Online inclusive and how they think PACT Online could best be implemented outside a trial. Analysis will be led by two independent qualitative researchers, drawing from reflexive thematic analysis principles.^52^

### Outcomes and significance

It is anticipated that this project will have a lasting impact on the health and well-being of children with NDD and their families through the delivery of an innovative online allied health intervention targeting the parent–child relationship and parenting. If successful, *PACT Online* will be sustainably embedded in existing health services for families of children with NDD, growing parent empowerment and better supporting parents, with long-term flow on effects for parent and child.

## Ethics and dissemination

Ethical approval has been obtained from the University of Queensland, Human Research (Ethics Approval 023/HE000040) and the study has been registered with the Australian New Zealand Clinical Trials Registry (ACTRN12623000612617) since 5 June 2023. PACT Online will be offered to the CAU group after T3 follow-up for ethical reasons and to maintain participant engagement. As a consequence, testing downstream benefits beyond 6 months will not be possible. Dissemination of study outcomes will occur through published papers in scientific journals, presentations at conferences and to study participants through the study update. If *PACT Online* is effective, the *PACT Online* intervention will be released as a massive open online course. The implementation analysis will result in the identification of contextual factors influencing implementation and the development of tailored implementation strategies.

Should the intervention be effective, this trial will contribute crucial evidence to support implementation of the *PACT Online* intervention for families of children with a disability, including informing cost-effectiveness through our health economic analysis. This is supported by our implementation plan and partners in the community, which will facilitate rapid uptake of findings and cross-sector collaborations between primary healthcare professionals, families of children with NDD and health policymakers.

## Supplementary material

10.1136/bmjopen-2024-088981 online supplemental file 1

10.1136/bmjopen-2024-088981 online supplemental file 2
