# Supplementary material for: Parenting Acceptance and Commitment Therapy Online (PACT Online) for parents of children diagnosed with or with increased likelihood of neurodevelopmental disability: study protocol of a randomised controlled trial
Source: BMJ Open. 2025 Jun 20;15(6):e088981. doi: 10.1136/bmjopen-2024-088981 (PMC12182011; doi:10.1136/bmjopen-2024-088981)
Supplement: online supplemental file 1 [file bmjopen-15-6-s001.docx]

**PACT Online Semi-Structured Interview**

Thank you for agreeing to give some verbal feedback about PACT Online. We are interested in hearing your thoughts and experiences in completing the PACT Online intervention so that it can be continued to be refined.

**Building the relationship and opening the story**

1. Tell me about yourself and your family (take as long as you like we want to get to know you better)
2. What was it about PACT Online that made you want to sign up to the study?
3. Did you have any reservations or hesitations in signing up for the PACT Online study? Tell me more about those reservations.

**The PACT Online Program: Overall experiences**

1. Tell me about your experience of participating in PACT Online? (take as long as you like we want to get to know you better)
2. What were the best parts of PACT Online? What worked well for you and your family?
3. What were the worst parts of PACT Online? What didn’t work well for you and your family?
4. What were the most difficult parts of PACT Online even if they might also have been helpful?
5. How flexible and adaptable was PACT Online to the needs of your child?
6. What did you think of format of PACT Online (online with zoom consultations)? Would you change anything regarding the format of the program?
7. What did the sessions with the clinicians add to the online modules, if anything?
8. Do you have anything further to add about the PACT Online program?

**The PACT Online Program: Legacy**

1. In the short term, what did you get out of taking part in the PACT Online program? What do you plan on continuing long-term?
2. What changes, if any, have you noticed in your child after doing PACT Online?
   1. What changes, if any, have you noticed in yourself after doing PACT Online?
   2. What about changes in yourself beyond parenting?
   3. How have relationships changed, if at all?
   4. How sustainable are these changes, from your perspective?
3. What aspects of PACT Online and the way we delivered it helped PACT Online to work for you and your family (if it did)?
4. Have you applied anything from PACT Online to your day-to-day life?
5. What support do you need to keep applying PACT Online in your day-to-day life?
6. Did any other family members (e.g. other parent, grandparents) take part in PACT Online? In what way did they take part?

a. What do you think they took away from the experience?

b. What was that like for you to have them taking part in PACT online?

**Future of the PACT Online program**

1. Who do you think PACT Online is best for?
2. Do you think that other parents of children *[with X NDD diagnosis]**  would benefit from PACT Online? If so, what makes you think that?
3. Do you think PACT Online should be promoted to other parents? If so, do you have any ideas on how?
4. Was PACT Online inclusive to diverse families? If not, how could we make PACT Online more inclusive?
5. What are your views on PACT Online being implemented within the health, disability and/or educational support systems?
   1. Reflecting on your experiences navigating these systems, what do you think will be important at a systems level to make that work? `
6. Thinking about how we could make the program work for you and have a long-term impact, are there any adaptations or changes you would have liked to see in order to meet your needs?
7. If PACT Online was implemented outside a trial, what barriers do you think we would have to address to make it more accessible to families? Think about both individual and systems (healthcare, social care, education) barriers.
8. What aspects of your context, who you are as a parent and as a person have helped/made it difficult for PACT online work for you and your family?
9. I now want you to reflect on your journey parenting your child and navigating the systems you have navigated as part of that journey (e.g., health, education, etc). At what point of that journey do think it would have been good to engage in a program like PACT online?

**Conclusion**

1. Is there anything else you would like to add?

*[with X NDD diagnosis]* - use the specific diagnosis appropriate for that child. For autism and ADHD, identify first language is usually preferred and should be used unless the parent is using person first language. In general, use the wording used by the parent in the interview.*

Thank you very much for your time.
