# Supplementary material for: Parenting Acceptance and Commitment Therapy Online (PACT Online) for parents of children diagnosed with or with increased likelihood of neurodevelopmental disability: study protocol of a randomised controlled trial
Source: BMJ Open. 2025 Jun 20;15(6):e088981. doi: 10.1136/bmjopen-2024-088981 (PMC12182011; doi:10.1136/bmjopen-2024-088981)
Supplement: online supplemental file 2 [file bmjopen-15-6-s002.docx]

Parent Participant Information Sheet

**Project Title Randomised Trial of Parenting Acceptance and Commitment Therapy for Parents of children diagnosed with or with increased chance of neurodevelopmental disability or developmental delay**

**HREC Number 2023/HE000040**

**Chief Investigators:** Dr Koa Whittingham, Prof Roslyn Boyd, Prof Iona Novak, Dr Amy

Mitchell Dr Natasha Reid, Dr Syed Afroz Keramat, A/Prof Kristelle Hudry, A/Prof Josephine

Barbaro, Dr Jacqueline Barfoot, A/Prof Rebecca Olson, Dr Nathalia Costa

**Associate Investigators**: Prof Robert Ware, Dr Fiona Russo, A/Prof Helen Heussler, Dr

Andrea McGlade, Dr Ashleigh Bullot, Megan MacDonald, Dr Tommy Tran, Sophie Harrington,

Dr Jeanie Sheffield

**Partner Organisations:** AEIOU Foundation, BUSHKids, Cerebral Palsy Alliance, Paeds in a

Pod, NOFASD.

**Research Staff:** Dr Grace Kirby (Research Trial Coordinator)

**Version Number: 2.2 Version Date: 17^th^ October 2024**

#### Thank you for taking the time to read this Parent Information Statement and Consent Form. We would like to ask you to participate in a research project that is explained below.

**It is ok to say no**

**If you would like a member of the research team to support you through this information and consent form, you can email us at** [**pactonline@uq.edu.au**](mailto:pactonline@uq.edu.au) **and we would be happy to organise a time for a phone call or online video call.**

**Summary of research**

- Free online parenting program for parents of children aged 0-10 years with a neurodevelopmental disability (e.g., cerebral palsy, autism and FASD) or developmental delay
- 12-week online program consisting of one self-paced module per fortnight
- One 30-60 minute telehealth consultation (via Zoom) per fortnight with therapist
- Completion of assessment items at three time points – before program, after program and at a 6-month follow up
- **Optional** additional assessment monitoring heart rate variability

**What is an Information Statement?**

These pages tell you about the research project. It explains to you clearly and openly all the steps and procedures of the project. The information is to help you decide whether or not you would like to take part in the research. Please read this Information Statement carefully.

Before you decide if you want to take part or not, you can ask us any questions about the project. You may want to talk about the project with your family, friends, or healthcare worker. Feel free to talk about the research project with someone who can help you get a full understanding of what will happen if you say yes to participating in the PACT Online research project.

**Important things to know**

- It is your choice whether or not you take part in the research. You do not have to agree if you do not want to.
- If you decide you do not want to take part, it will not affect the treatment and care you receive through your local clinical service or any of our partner organisations.

If you would like to take part in the research project, please sign the consent form provided by the Researcher. By signing the consent form you are telling us that you:

- understand what you have read
- had a chance to ask questions and received satisfactory answers
- consent to take part in the project

We will give you a copy of this information and consent form to keep.

#### There are 9 pages of this information sheet. Please check to see if you have all the pages.

#### Body of Information Sheet:

#### What is the research project about?

In previous research, parents of children with diagnosed with a neurodevelopmental disability (NDD; including cerebral palsy, autism and FASD) have told us that they want parenting support that is flexible and specific to their needs. We (the PACT Online Investigators) **listened**, and we developed PACT Online. PACT Online is an online parenting support program including an online course and telehealth sessions with an experienced therapist. It is grounded in a form of cognitive behavioural therapy called acceptance and commitment therapy (ACT) and it is focussed on supporting you in being the kind of parent that **you want to be** while **taking care of yourself** as well.

**Can I participate?**

If you are the parent of a child 0-10 years of age with a diagnosis of any NDD (including autism, cerebral palsy, FASD, intellectual impairment, ADHD) or a diagnosis of developmental delay (across two or more domains or global developmental delay) or assessed as increased chance for cerebral palsy using the GMA or identified as increased likelihood for autism using the SACS then you can participate in PACT Online. Note, we consider you a parent if you are fulfilling a **parental role** including step and foster parents and grandparents who are the child’s primary caregiver. We will be using the word ‘parent’ in this way throughout this study.

**What is involved in the study?**

If you provide your consent, you have the right for your information to be treated confidentially. You also have a responsibility to do your best to meet the commitments of the online research study program.

Because we need to compare the new intervention to something else, we have 2 groups. One group gets the online intervention right away (NOW), and one group gets put on a wait list (WAIT). Families will be randomly (like the flip of a coin) assigned to one of the 2 groups. Families who are on the wait list will still get to have access to the online intervention at the end of six months of waiting. Having one group who receives PACT Online immediately and one group who continues with routine regular care will help us to understand whether PACT Online leads to better outcomes.

**What do I need to do in this research project?**

**Firstly, you will need to participate in the PACT Online intervention** when it is your turn (either now or in six months’ time).

The PACT Online intervention takes 12 weeks. It includes an online course using EdX and telehealth sessions with an experienced therapist. The online course includes videos, text, online activities, and opportunities to apply the content in your daily life. Involvement in the online course is very flexible and the course has been designed to minimise parent burden. You will move through one module per fortnight (at your pace and using a computer, smartphone or tablet). Each module takes 1-2 hours to complete. In addition, you will receive one 30-60 minute telehealth consultation (via Zoom) per fortnight, to support you in understanding and acting on that fortnight’s module. During PACT Online you’ll be supported in making and moving towards your own personalised goals for change: (1) a goal for your relationship with your child or a parenting goal, (2) a goal for your own self-care or health, and (3) a goal for your child’s behaviour or adjustment.

The telehealth sessions with your therapist will be recorded. This is all part of ensuring that we as a team are sticking to our research plan and is usual for a project like this.

**Secondly, you will need to complete the assessments** when it is your turn. You will need to complete assessments three times during this study. All assessments will be conducted online or in your own home.

You will be asked to complete questionnaires online. The questionnaires will ask you about your child’s disability and behaviour, your own wellbeing and adjustment and your family. The questionnaires will take approximately an hour to complete so it will be important to set aside some time to do them. We can support you with a Zoom or a phone call in order to achieve this.

In addition, a time will be made for us to make a videorecording of you interacting with your child in your own home. This will be recorded using Zoom and should be an ordinary interaction with your child. We will support you in setting this up.

For some participating families, you may also be sent a monitor to take measurements of your and your child’s heart rate variability. Heart variability is the fluctuations in the amount of time between heartbeats and it is a good measure of stress. Measuring your and your child’s heart rate variability is simple and will take five minutes. We will send full instructions with the monitor and be in touch to support you to do this. If you do not wish to take part in this part of the study to monitor heart rate variability that is okay. You may choose to opt out of this part of the assessment and still complete the rest of the study as normal.

A List of the Assessments involved in PACT Online is attached at the end of this Information sheet. Feel free to ask questions about any of the assessments.

**Are there any risks to participation?**

This study contains no risks beyond everyday living. PACT Online will explore your values, your feelings and thoughts and your general well-being, in order to support you in being the kind of parent that you want to be and living the life you want to live. At times this may challenge you. Your experienced therapist will be able to support you. You are free to share only what you feel comfortable sharing. You can always just say no, or not this time.

**Will my information be confidential?**

Yes! All information that we collect from you including your questionnaires, video recordings, assessments and your feedback will be stored in a confidential manner on a database at the Queensland Cerebral Palsy and Rehabilitation Research Centre, South Brisbane Queensland. The forms containing assessment or questionnaire results will be kept until the end of the study and will then be held at the Queensland Cerebral Palsy and Rehabilitation Research Centre in Brisbane in a code-locked computer file. The information in this study will only be used in ways that will not reveal who you are. You will not be identified in any publication from this study or in any data files shared with other researchers. All information will be held in strict confidence and will be used for statistical purposes only. Data collected from you will be de-identified, by replacing any identifying information (e.g., your name) with a participant number. Deidentified data may be archived in accordance with current best practices in research. Identified or de-identified research data may be made available for review by ethics review committees or other regulatory authorities for the purposes of monitoring the ethical and scientific conduct of the study. We will only share your personal information if you have given us consent to or if we are required or authorised to disclose your information by law. One important exception to confidentiality is if we have reasonable suspicion of abuse or neglect as in those circumstances, we are ethically obligated to contact Child Safety Services.

**What if I change my mind?**

You do not have to take part in this research project. Your participation will not affect any treatment that you receive. If you do agree to participate, or if you agree and then change your mind at a later date, you are free to withdraw from the study at any time without any negative consequence. You can withdraw from the study at any time by completing and signing the ‘Participant Withdrawal of Consent Form’. This form is provided at *the end of this document*, and is to be completed by you and supplied to the research team if you choose to withdraw at a later date.

If you withdraw from the study, you will be able to choose whether the study will destroy or retain the information it has collected about you. You should only choose one of these options. Where both boxes are ticked in error or neither box is ticked, the study will destroy all information it has collected about you.

**Who is involved?**

**Chief Investigators**

1. **Dr Koa Whittingham** is a clinical and developmental psychologist and a senior research fellow at the University of Queensland. She is experienced in working with parents of children with neurodevelopmental disabilities, as well as working with ACT, and is a developer of the PACT Online intervention.
2. **Professor Roslyn Boyd** is the Scientific Director of Queensland Cerebral Palsy and Rehabilitation Research Centre. She has clinical and scientific expertise in working with children with cerebral palsy and developmental delay and their families.
3. **Prof Iona Novak** is the Cerebral Palsy Alliance Chair of Allied Health, The University of Sydney. Professor Iona Novak is an occupational therapist and Fulbright Scholar focused on conducting a research program of neuro-protective, neuro-regenerative and neuro-rehabilitative clinical trials in cerebral palsy.
4. **Dr Amy Mitchell** is a Senior Lecturer, School of Nursing, Midwifery and Social Work and Honorary Research Fellow in the Parenting and Family Support Centre, The University of Queensland. She is a paediatic nurse with expertise in supporting parents and families of children with special health and developmental needs.
5. **Dr Natasha Reid** is a Research Fellow & Clinical Psychologist, Child Health Research Centre, Faculty of Medicine, The University of Queensland. Dr Reid is a recognised national and emerging international expert in fetal alcohol spectrum disorder.
6. **Dr Syed Afroz Keramat** is a Postdoctoral Research Fellow with the Centre for Health Services Research, Faculty of Medicine, The University of Queensland. Dr Afroz completed his PhD in health economics at the University of Southern Queensland and will be contributing his expertise to the economic evaluation of PACT Online.
7. **Dr Kristelle Hudry** is a Senior Lecturer within the Department of Psychology and Counselling at the La Trobe University. She is experienced in working with parents of children with Autism Spectrum Disorder.
8. **A/Prof Josephine Barbaro** is a Principal Research Fellow and Registered Psychologist, Olga Tennison Autism Research Centre (OTARC), La Trobe University. A/Prof Barbaro is an international expert in early detection and diagnosis of autism, developing the Social Attention and Communication Surveillance (SACS) during her PhD.
9. **Dr Jacqui Barfoot** is an Occupational Therapist with clinical and research experience in parent-infant relationship focussed interventions to support infant development.
10. **A/Prof Rebecca Olson** is an experienced qualitative researcher with expertise in conducting qualitative research in an implementation science context.
11. **Dr Nathalia Costa** is an experienced qualitative researcher with expertise in conducting qualitative research in an implementation science context.

**Associate Investigators**

1. **Prof Robert Ware** is a Biostatistician with >130 peer reviewed publications and extensive experience in clinical environments will provide expert advice and oversee all analyses.
2. **Dr Fiona Russo** is our Consumer Coordinator. Dr Russo will act as a Consumer Coordinator, leading the consumer engagement panel. As a researcher and a parent of a child with NDD with training in consumer engagement she is uniquely qualified for this role.
3. **A/Prof Helen Heussler** is a Partner Investigator and Paediatrician. A/Prof Heussler will coordinate any recruitment through Queensland Health.
4. **Dr Andrea McGlade** is a Partner Investigator and Paediatrician. Dr McGlade will assist in managing recruitment through Paeds in a Pod.
5. **Dr Ashleigh Bullot** is a Partner Investigator, Psychologist and Head of Resaerch at AEIOU. Dr Bullot will assist in managing recruitment through the AEIOU Foundation.
6. **Megan MacDonald** is a Partner Investigator and lead occupational therapist at BUSHKids. Ms MacDonald will assist in managing recruitment through BUSHkids
7. **Dr Tommy Tran** is a Partner Investigator and Paediatrician. Dr Tran will assist in managing recruitment through Paeds in a Pod.
8. **Sophie Harrington** is a Partner Investigator and parent. Ms Harrington will assist in managing recruitment through NOFASD.
9. **Dr Jeanie Sheffield** is a Psychologist and, alongside Dr Koa Whittingham, a devleoper of PACT Online. Dr Sheffield will support in any tailoring of PACT Online throughout and support in fidelity.

**Consumer Engagement**

A consumer engagement panel will be led by Dr Russo (parent of a child with NDD and early career researcher) to ensure good consumer engagement throughout.

**Support**

If you become distressed while participating in this study support is available through Lifelife at <https://www.lifeline.org.au/> or by phoning 13 11 14 or through Beyond Blue at https://www.beyondblue.org.au/ or by phoning 1300 22 4636.

**Do you have any questions?**

Please take the time to ask us any questions that you may have. You can contact:

**Dr Grace Kirby (Study Coordinator) or Dr Koa Whittingham (Chief Investigator A)**

on (07) 3443 6395

or email [pactonline@uq.edu.au](mailto:pactonline@uq.edu.au)

**University of Queensland Ethics Contact:**

This study adheres to the Guidelines of the ethical review process of The University of Queensland and the National Statement on Ethical Conduct in Human Research. Whilst you are free to discuss your participation in this study with project staff, if you would like to speak to an officer of the University not involved in the study, you may contact the Ethics Coordinators on (07) 3365 3924 / (07) 3443 1656, or email [humanethics@research.uq.edu.au](mailto:humanethics@research.uq.edu.au).

**PARTICIPANT CONSENT FORM**

**Project Number:**

**Project Title PACT Online: Randomised Trial of Parenting Acceptance and Commitment Therapy for Parents of children with neurodevelopmental disabilities**

**Chief Investigators** Dr Koa Whittingham, Prof Roslyn Boyd, Prof Iona Novak, Dr Amy Mitchell Dr Natasha Reid, Dr. Syed Afroz Keramat, A/Prof Kristelle Hudry, A/Prof Josephine Barbaro, Dr. Jacqueline Barfoot, A/Prof Rebecca Olson, Dr Nathalia Costa.

- I have read the information statement for this study and I believe I understand the purpose, extent and possible effects of my involvement.
- I have had an opportunity to ask questions and I am satisfied with the answers I have received.
- I understand information collected will be stored confidentially and my identity will not be revealed unless I give permission for information to be shared or it is required by law.
- I understand that I can refuse to participate and can withdraw from this study at any time without any negative consequence. In particular, I understand that my participation will not affect my access to usual medical care.
- I understand that the purpose of this study is an innovative, online approach to early intervention and that my active involvement, including critical feedback, is valued.
- I understand that in order to evaluate the new early intervention I will be asked to complete online questionnaires as well as record an ordinary parent-child interaction during the study.
- **I consent to participate in this research project.**

**OPTIONAL – Further Research**

- I would like to be contacted about any future research trials that I may be eligible to participate in. (NB. full ethical approval would be sought by the research team and a new consent process undertaken) **Yes / No**

I voluntarily consent to participate in the above titled Research Project explained to me by:

Ms/Dr/Professor _______________________________________________

**Participant’s Name ________________________________ Date ____________________**

**Participant’s Signature ________________________________**

**List of Assessments**

**Questionnaires**

Most of the assessments are questionnaires and will be done online via REDCap. The questionaries will include asking about:

- **Your family and your child** using a demographic questionnaire and the Gross Motor Function Classification System (GMFCS). The GMFCS is a parent-rated measure of gross motor ability, particularly important for families of children with a physical disability.
- **Your relationship with your child** including the Emotional Availability Self-Report and the Interpersonal Mindfulness in Parenting Scale.
- **Your own adjustment, health behaviour and wellbeing**. This will include the Depression Anxiety Stress Scales (DASS-21), the Parenting Empowerment and Efficacy Measure (PEEM), the Good Health Practices Scale, Parent Experience of Child Illness Scale (disability version), the EQ-5D-5L (health related quality of life), the Carer Experience Scale (CES) and the Comprehensive Assessment of Acceptance and Commitment Therapy Processes (ComPACT).
- **Your child’s disability, adjustment and wellbeing.** This will include the Vineland Adaptive Behaviour Scale Comprehensive (VABS-III) and the Child Behaviour Checklist (CBCL). You’ll also be asked about any medical visits related to the child’s NDD, any interventions you accessed for your child during the study and the impact of this on you.
- **Your goals.** We will also ask you about your goals in PACT Online using the Goal Attainment Scale.

**An interaction with your child**

We will also record an ordinary interaction between you and your child in your own home. We will do this via Zoom. This is to help us get a better understanding of your relationship with your child. We will look at this interaction using the Emotional Availability Scales.

**Heart rate variability**

Heart variability is a measure of the fluctuations in the amount of time between heartbeats and it is a good measure of stress. Measuring your and your child’s heart rate variability is simple and will take five minutes. We will send full instructions with the monitor and be in touch to support you to do this.

**Feedback**

It is important to us to have feedback on PACT Online. After you have participated in PACT Online we will ask if you are able to give us feedback. If you are, we will make a time to have a Zoom call with you.

**Participant Withdrawal of Consent Form**

**Project Title Randomised Trial of Parenting Acceptance and Commitment Therapy for Parents of children diagnosed with or with increased chance of neurodevelopmental disability or developmental delay**

**HREC Number 2023/HE000040**

I wish to withdraw my consent to participate in this study effective from the date below.

I would like my already collected and unanalysed data (please choose one option):

Destroyed and no longer used for research

Retained and continued to be used for research

I understand that:

- I will not be asked to provide any more information from the withdrawal date listed below
- Information already analysed and/or included in a publication by the study may not be able to be destroyed
- Withdrawal will not affect my relationship with the University of Queensland, or any other organisation or professionals listed in the Parent Participant Information Sheet

**……………………………………………… …………………………**

**Signature Date**

**…………………………………………………………………………………………**

**Please print full name**

**Please email this form to:** [**pactonline@uq.edu.au**](mailto:pactonline@uq.edu.au)

**Alternatively, forms can be posted to**: Dr Koa Whittingham, Queensland Cerebral Palsy and Rehabilitation Research Centre, The University of Queensland, Level 6, 62 Graham Street, South Brisbane QLD 4101
