# Supplementary material for: Parenting Acceptance and Commitment Therapy Online (PACT Online) for parents of children diagnosed with or with increased likelihood of neurodevelopmental disability: study protocol of a randomised controlled trial
Source: BMJ Open. 2025 Jun 20;15(6):e088981. doi: 10.1136/bmjopen-2024-088981 (PMC12182011; doi:10.1136/bmjopen-2024-088981)
Supplement: Supplementary file 3 [file bmjopen-2024-088981.reviewer_comments.pdf]

PEER REVIEW HISTORY

BMJ Open publishes all reviews undertaken for accepted manuscripts. Reviewers are asked to complete a checklist review form and are provided with free text boxes to elaborate on their assessment. These free text comments are reproduced below.

ARTICLE DETAILS

Title (Provisional)

Parenting Acceptance and Commitment Therapy Online (PACT Online) for parents of children diagnosed with or with increased likelihood of neurodevelopmental disability: Study protocol of a randomised controlled trial

Authors

Whittingham, Koa; Kirby, Grace; Boyd, Roslyn N; Novak, Iona; Mitchell , AE; Reid, Natasha; Keramat, Afroz; Hudry, Kristelle; Russo, Fiona; Bullot, Ashleigh; MacDonald, Megan; Tran, Tommy; Harrington, Sophie; Barbaro, Josephine; Barfoot, Jacqui; Ware, Robert S.; Heussler, Honey; McGlade, Andrea; Sheffield, Jeanie; Olson, Rebecca; Costa, Nathalia

---

VERSION 1 - REVIEW

**Reviewer**	**1**
**Name**	**Liu, Guihua**
**Affiliation**	**Fujian Provincial Maternity and Children's Hospital**
**Date**	**28-Dec-2024**
**COI**	**None**

Thank you for inviting me to review this protocol of a randomised controlled trial, which investigates the effect of Parenting Acceptance and Commitment Therapy Online (PACT Online) for parents of children diagnosed with or with increased likelihood of neurodevelopmental disability.

1. In the Abstract, “This project aims to assess the effectiveness and implementation of an online parenting support program, PACT Online, for parents of children with neurodevelopmental disabilities for improving the parent-child relationship and parent and child outcomes.” However, this article focuses more on evaluating parent-child relationships and parents' mental health. Is it appropriate to describe the impact on children here?

2. Different types of neurodevelopmental disability vary greatly, and their psychological impacts on parents are also different. Is it appropriate to include all of them? Is hierarchical analysis necessary? This is something that the author needs to focus on and think about.

3. Is software used for sample size calculation? What software is used? “To detect a difference of 0.35SD on the Emotional Availability Scales”, what it is based on should be clearly explained.

4. The intervention measures for the control group should also be described in detail to make them reproducible.

5. There are many questionnaires. How to ensure the participation and quality of questionnaire evaluation?

6. The reliability and validity of the scale in this study need to be considered.

7. How is the 12-week EdX course implemented and what is the frequency of intervention?

**Reviewer**	**2**
**Name**	**Pontoppidan, Maiken**
**Affiliation**	**VIVE, Health**
**Date**	**09-Jan-2025**
**COI**	**None**

Title: Parenting Acceptance and Commitment Therapy Online (PACT Online) for Parents of Children Diagnosed with or with Increased Likelihood of Neurodevelopmental Disability: Study Protocol of a Randomised Controlled Trial

Thank you for the opportunity to review this well-conceived study protocol. The introduction is well-written, and the topic is highly relevant for both research and clinical practice. However, I have some points where further clarification and additional details would strengthen the protocol:

Study Registration:

Please provide the exact date of registration and a direct link to the trial registry for transparency and reproducibility.

Recruitment:

Could you clarify the recruitment period, including specific start and end dates?

Sample Size:

Please elaborate on the rationale behind the choice of a 0.35 SD on the EAS as the expected effect size. How was this effect size determined?

Randomization:

The randomization process requires further details. How will the recruitment flow work? Will participants be randomized immediately after consent, or will there be a pre-

randomization screening? Clarifying the sequence of steps will improve the protocol's transparency.

Intervention:

Could you expand on the theoretical foundation of the PACT Online intervention?

Who developed the intervention, and has it been tested in previous trials except from your pilot?

How many sessions does the intervention consist of, and what is the typical duration?

Is the intervention delivered in groups or individually?

I was unable to locate the intervention on the EDX platform.

Outcome Measurement:

This section primarily describes questionnaire-based data collection. I recommend also including a description of the observational data being collected here.

What guidelines are parents given for the observational tasks? Can toys be used during the observation?

Are there different guides according to child age (e.g., it can be difficult to film 1- and 2-year olds if they are walking around playing)?

For infants and toddlers, 20 minutes may be lengthy.

How do you apply the EAS coding across the broad age range (0-10 years)? Are the same items and constructs being coded for all children, or will adjustments be made based on the child's age?

Is the EAS global score the primary outcome, with subscales serving as secondary outcomes? Clarifying this distinction would be helpful.

Questionnaire Length and Completion:

The questionnaire appears quite extensive. How long is the estimated completion time?

What strategies will you use to ensure parents complete the questionnaires at post-intervention and follow-up?

Interviews:

Could you provide more details on the qualitative interviews with both parents and clinicians?

What are the primary themes and example questions?

How do you plan to analyze the qualitative data?

Subgroup Analyses:

Please clarify how the subgroup analyses based on child age and diagnosis will be conducted. How will the groups be divided for analysis?

Imputation:

The imputation strategy needs further clarification. What do you mean by "Imputation techniques will be used to test the sensitivity of results to missing data if necessary"? Under what conditions will imputation be considered necessary?

VERSION 1 - AUTHOR RESPONSE

Reviewer: 1 Mrs. Guihua Liu, Fujian Provincial Maternity and Children's Hospital

Thank you for inviting me to review this protocol of a randomised controlled trial, which investigates the effect of Parenting Acceptance and Commitment Therapy Online (PACT Online) for parents of children diagnosed with or with increased likelihood of neurodevelopmental disability.

1. In the Abstract, "This project aims to assess the effectiveness and implementation of an online parenting support program, PACT Online, for parents of children with neurodevelopmental disabilities for improving the parent-child relationship and parent and child outcomes." However, this article focuses more on evaluating parent-child relationships and parents' mental health. Is it appropriate to describe the impact on children here?

Our response: Yes, it is. The impact on children is included in the secondary outcomes including child mental health as measured by the Child Behaviour Checklist, child adaptive behaviour as measured by the Vineland and child regulatory abilities as measured by heart rate variability.

2. Different types of neurodevelopmental disability vary greatly, and their psychological impacts on parents are also different. Is it appropriate to include all of them? Is hierarchical analysis necessary? This is something that the author needs to focus on and think about.

Our response: Thank-you. While neurodevelopmental disabilities vary, there is also significant variation within neurodevelopmental disabilities in terms of child outcomes, prognosis and impact on parents. The major advantage of developing and trialling an intervention for all neurodevelopmental disabilities is that such an intervention is more likely to be readily disseminated. This same approach has been used with success for other programs, for example, Stepping Stones Triple P. We intend to do a sub-group analyses to compare outcomes by child age and diagnosis of neurodevelopmental disability and this is specified in the section on statistical analysis.

3. Is software used for sample size calculation? What software is used? "To detect a difference of 0.35SD on the Emotional Availability Scales", what it is based on should be clearly explained.

Our response: The statistical software used was Stata statistical software v14 (StataCorp, College Station, TX, USA). We have included this information in the manuscript. We have also clarified how a difference of 0.35SD was determined. The text now reads:

"The sample size calculation is based on the between-group difference for the primary outcome, the global relationship quality score from the EAS, which ranges from 0 (worst relationship possible) to 100 (best relationship possible). Based on a previous RCT conducted by our team in 67 families of children with a neurodevelopmental disability (cerebral palsy) we assume the standard deviation (SD) of the Global EAS will be 14.3 units, and that identifying a difference between the active (PACT) and waitlist control group of 5.0 units is clinically important. This is equivalent to a between-group difference of 0.35SD. To achieve 80% power, 258 families (129 in each group) are required to provide outcome data (alpha=0.05). To allow for expected 11-15% participant attrition (as experienced in our previous trials; (25) we will recruit a total of 300 families into this RCT. Participant retention strategies will be guided by the consumer engagement panel and will include a quarterly study update. Sample size calculations were conducted using Stata statistical software v14 (StataCorp, College Station, TX, USA)."

4. The intervention measures for the control group should also be described in detail to make them reproducible.

Our response: Thank-you. The PACT Online and the CAU control group participants will both completed T1, T2 and T3 assessments. That is, the measures are the same for both groups.

5. There are many questionnaires. How to ensure the participation and quality of questionnaire evaluation?

Our response: We acknowledge that there are many questionnaires, and we are grateful to the parents for taking the time to complete them. The questionnaires take approximately one hour to complete in total and we have added this clarity to the manuscript. One critical way that we ensure participant engagement and "repay" participants for the time taken in assessment is giving all participants the PACT Online intervention. That is, the CAU control

families are given PACT Online after they complete their T3 follow up assessment. The families understand that they will all receive an intervention for free by participating and we find that this goes a long way to encouraging their willingness to complete the questionnaires. In addition, we send all participants a quarterly study update and have received input on our assessment procedures, including assessment burden, from our consumer engagement panel. This helped us to make questionnaire completion as straight forward as possible.

6. The reliability and validity of the scale in this study need to be considered.
Our response: We do comment on the reliability and validity of our measures throughout. For example, for the EA-SR we state, "Good reliability estimates for the subscales have been found ranging from .71 and .84, with the exception of the affect quality subscale (α=.49). The EA-SR has excellent validity and has been validated against the EAS.". If there is a particular measure that needs additional information, we would be guided by the Editor in this and happy to provide it.

7. How is the 12-week EdX course implemented and what is the frequency of intervention?
Our response: Thank-you. We've tweaked our description of the PACT Online intervention for clarity and detail, including in terms implementation and frequency of sessions. It now reads as below:

"PACT ("Parenting with Acceptance and Commitment Therapy") *Online* is an online/telehealth parent support intervention developed in partnership with parents and focused on empowering parents as well as supporting parenting, parental mental health, and the parent-child relationship. It is theoretically grounded in acceptance and commitment therapy and was developed by authors Dr Koa Whittingham and Dr Jeanie Sheffield. PACT Online as tested in this RCT is an enhanced and adapted intervention of PACT as tested in an RCT with 68 families of children aged 2-12 years with CP
PACT Online is delivered over 12 weeks via the edX platform (www.edx.org/) and six telehealth sessions. It includes core ACT content and adopts a flexible, goal-directed approach focusing on each parent's tailored behavioural goals. Parents complete six modules over 12 weeks, a module per fortnight, with an individual telehealth session for each module. In the individual telehealth sessions trained allied health clinician provide individualised feedback and additional support, helping families to apply the content to their lives. *PACT Online* thus offers a translatable and scalable universal online support at little to no cost, with additional

clinician-delivered telehealth support for families who need it.”

Reviewer: 2 Dr. Maiken Pontoppidan, VIVE
Thank you for the opportunity to review this well-conceived study protocol. The introduction is well-written, and the topic is highly relevant for both research and clinical practice. However, I have some points where further clarification and additional details would strengthen the protocol:
Study Registration:
Please provide the exact date of registration and a direct link to the trial registry for transparency and reproducibility.
Our response: I believe we’d removed the link for blinding, but we have ensured it is included now along with the date of registration, 5/6/2023. For ease of reference, the registration number is: ACTRN12623000612617.

Recruitment:
Could you clarify the recruitment period, including specific start and end dates?
Our response: Recruitment began on 26/02/2024 and it is planned to continue until approximately mid-2026. We cannot specify an exact end date as recruitment is still in progress. We have included the recruitment start date and planned end date in the manuscript under the recruitment section.

Sample Size:
Please elaborate on the rationale behind the choice of a 0.35 SD on the EAS as the expected effect size. How was this effect size determined?
Our response: Thank you for this comment, which is very similar to a question by Reviewer 1. Please see our response above for the revised sample size paragraph.

Randomization:
The randomization process requires further details. How will the recruitment flow work? Will participants be randomized immediately after consent, or will there be a pre-randomization screening? Clarifying the sequence of steps will improve the protocol's transparency.
Our response: Thank-you. The randomisation will occur after screening to ensure eligibility, consent and the completion of the baseline assessment. We have edited the section on randomisation and blinding to add this clarity. It now reads: “Following screening to ensure

eligibility, consent and the completion of baseline assessment, families will be randomised to *intervention* or CAU using central computer-generated block randomisation (Griffith University)."

Intervention:

Could you expand on the theoretical foundation of the PACT Online intervention?

Who developed the intervention, and has it been tested in previous trials except from your pilot?

How many sessions does the intervention consist of, and what is the typical duration?

Is the intervention delivered in groups or individually?

I was unable to locate the intervention on the EDX platform.

Our response: Thank-you. We have edited the section on the intervention to provide further details and clarity as suggested. Please note that PACT Online is not currently publicly available on the EdX platform because it is still under trial. When the trial is completed, if it is successful, then it will be publicly available. The section on the intervention now reads as follows:

"PACT ("Parenting with Acceptance and Commitment Therapy") *Online* is an online/telehealth parent support intervention developed in partnership with parents and focused on empowering parents as well as supporting parenting, parental mental health, and the parent-child relationship. It is theoretically grounded in acceptance and commitment therapy and was developed by authors Dr Koa Whittingham and Dr Jeanie Sheffield. PACT Online as tested in this RCT is an enhanced and adapted intervention of PACT as tested in an RCT with 68 families of children aged 2-12 years with CP. *PACT Online* is delivered over 12 weeks via the edX platform (www.edx.org/) and six telehealth sessions. It includes core ACT content and adopts a flexible, goal-directed approach focusing on each parent's tailored behavioural goals. Parents complete six modules over 12 weeks, a module per fortnight, with an individual telehealth session for each module. In the individual telehealth sessions trained allied health clinician provide individualised feedback and additional support, helping families to apply the content to their lives. *PACT Online* thus offers a translatable and scalable universal online support at little to no cost, with additional clinician-delivered telehealth support for families who need it."

Outcome Measurement:

This section primarily describes questionnaire-based data collection. I recommend also including a description of the observational data being collected here.

What guidelines are parents given for the observational tasks? Can toys be used during the observation?
Are there different guides according to child age (e.g., it can be difficult to film 1- and 2-year olds if they are walking around playing)?
For infants and toddlers, 20 minutes may be lengthy.
How do you apply the EAS coding across the broad age range (0-10 years)? Are the same items and constructs being coded for all children, or will adjustments be made based on the child's age?
Is the EAS global score the primary outcome, with subscales serving as secondary outcomes? Clarifying this distinction would be helpful.
Our response: Thank-you. Yes, EAS global is the primary outcome. We have added details for the observational procedures and the Emotional Availability Scales as requested. The EAS section now reads as below:

Emotional Availability Scales (EAS) measures the quality of the parent-child relationship across six scales: parental sensitivity, parental structuring, parental non-intrusiveness, parental non-hostility, child responsiveness and child involvement, and a global relationship quality score. A 20-minute naturalistic observation of parent-child interaction in the home will be recorded via Zoom and subsequently coded by experienced and accredited EAS coders, kept blind to the intervention condition and timepoint. Parents will be advised that during the recording they should interact with their child in an ordinary, everyday way. For example, they could choose to play with toys, read a book, draw or perform any other interactive activity. Any child behaviour should be dealt with as they normally would. Extensive use of the EAS has demonstrated the excellent construct validity of this scale in establishing the expected relationship between EA and parent-child attachment. The EAS is appropriate for the full range of child ages in this study (0-10 years) with specific manuals guiding how to apply the same constructs for early (0-5 years) and middle (6-17 years) childhood. Further, our research group has achieved fair to excellent inter-rater reliability access the six scales in a similar study: parental sensitivity (ICC=0.72), parental structuring (ICC=0.51), parental non-intrusiveness (ICC=0.69), parental non-hostility (ICC=0.82), child responsiveness (ICC=0.82) and child involvement (ICC=0.71).”

Questionnaire Length and Completion:
The questionnaire appears quite extensive. How long is the estimated completion time?

What strategies will you use to ensure parents complete the questionnaires at post-intervention and follow-up?

Our response: We acknowledge that there are many questionnaires, and we are grateful to the parents for taking the time to complete them. The questionnaires take approximately one hour to complete in total and we have added this clarity to the manuscript. One critical way that we ensure participant engagement and "repay" participants for the time taken in assessment is giving all participants the PACT Online intervention. That is, the CAU control families are given PACT Online after they complete their T3 follow up assessment. The families understand that they will all receive an intervention for free by participating and we find that this goes a long way to encouraging their willingness to complete the questionnaires. In addition, we send all participants a quarterly study update and have received input on our assessment procedures, including assessment burden, from our consumer engagement panel. This helped us to make questionnaire completion as straight forward as possible.

Interviews:

Could you provide more details on the qualitative interviews with both parents and clinicians?

What are the primary themes and example questions?

How do you plan to analyze the qualitative data?

Our response: The qualitative interviews and the analysis will be conducted with an implementation lens and grounded in CFIR as a meta-theoretical framework. That is, the focus will be on understanding acceptability and appropriateness, contextual factors shaping implementation and informing the development of implementation strategies. The data will be analysed by reflexive thematic analysis. Additional details of the kinds of questions asked are now given and it now reads as follows:

"Qualitative analyses will be conducted on (i) semi-structured interviews with a subgroup of *PACT Online* participants and (ii) semi-structured interviews with therapists delivering PACT Online, (iii) semi-structured interviews with stakeholders in partner organizations, and (iv) a randomly selected sub-set of clinician notes. The CFIR, a meta-theoretical framework synthesising implementation theories, will be used to inform coding and analysis with a focus on ensuring that (i) *PACT Online* is acceptable and appropriate for parents, meeting their needs and (ii) the contextual factors shaping implementation in existing health, educational and disability services are understood, with appropriate implementation strategies developed. For example, parents will be asked what the best,

worst and most difficult parts of PACT Online were, what changes they've noticed, whether or not they found PACT Online inclusive and how they think PACT Online could best be implemented outside a trial. Analysis will be led by two independent qualitative researchers, drawing from reflexive thematic analysis principles."

Subgroup Analyses:
Please clarify how the subgroup analyses based on child age and diagnosis will be conducted. How will the groups be divided for analysis?
Our response: The exact subgroups used will depend upon the characteristics of the sample recruited. In terms of child age, we will compare older to younger children, and in terms of diagnoses we will group families into sub-groups of similar diagnoses, but the most logical grouping will depend on the ages of children recruited and the types and numbers of different diagnoses.
Imputation:
The imputation strategy needs further clarification. What do you mean by "Imputation techniques will be used to test the sensitivity of results to missing data if necessary"? Under what conditions will imputation be considered necessary?
Our response: Thank you. We have added more detail on our planned sensitivity analyses in the text:
"In the event that >10% of outcomes are missing, we will conduct a sensitivity analysis to investigate the sensitivity of results. The pattern of missingness will be examined, and if the data is 'missing at random' we will use multiple imputation by chained equations, whereas if the data is determined to be 'missing not at random' we will use pattern mixture models."

VERSION 2 - REVIEW

**Reviewer**	**1**
**Name**	**Liu, Guihua**
**Affiliation**	**Fujian Provincial Maternity and Children's Hospital**
**Date**	**25-Mar-2025**
**COI**	

General Assessment

This study protocol presents a Hybrid Type 1 RCT evaluating the effectiveness and implementation of PACT Online, an online parenting intervention grounded in Acceptance and Commitment Therapy (ACT) for parents of children with neurodevelopmental disabilities (NDD). The manuscript addresses a critical gap in accessible, tailored support for this population. The integration of effectiveness and implementation analyses, alongside a robust consumer engagement strategy, strengthens the potential for real-world impact. Below are specific comments and suggestions for improvement.

1. Why were families with children aged 0-10 years selected? What is the rationale for this age range?

2. Which family members participated - fathers, mothers, or both? The description in the text is unclear.

3. The inclusion criteria focus more on children. What are the inclusion conditions for parents?

4. Was the sample size calculation based on a pilot study? What software or formula was used for sample size calculation? The paper lacks a discussion section.

5. The description of the control group "Care-as-Usual (CAU)" is insufficient. Recommend adding detailed CAU descriptions (e.g., types of routine services, frequency) to clarify inter-group differences.

6. Only outcome assessors were blinded, while participants and intervention implementers were not, which may introduce performance bias. This limitation should be explicitly addressed in the discussion.

7. The plan to interview 30-40 parents lacks explanation of sampling strategy (e.g., maximum variation sampling) or data saturation assessment. Recommend adding justification for qualitative study design.

8. While the Good Health Practices Scale (GHPS) was used for parental health behaviors, there's no mention of its psychometric validation in NDD parent populations. Psychometric properties (reliability/validity) need documentation. The low internal consistency (α=0.49) of the "affect quality" subscale in EA-SR may affect result reliability, requiring researchers' attention.

9. While the study aims to recruit Australia-wide, there is limited discussion of strategies to ensure diversity (e.g., socioeconomic status, cultural/linguistic backgrounds). Given the exclusion of non-English-speaking families, the authors should address how this impacts generalizability and whether plans exist for future adaptations (e.g., translations).

10. The use of the Consolidated Framework for Implementation Research (CFIR) is appropriate, but the manuscript lacks specificity about which CFIR domains/constructs will guide qualitative data collection and analysis. A brief table or paragraph mapping CFIR constructs to interview questions/analysis goals would enhance clarity.

11. When using Zoom to record parent-child interactive videos, it is necessary to explain the data storage method (such as encrypted servers), access control, and video destruction plan to comply with ethical requirements.

12. The manuscript does not address potential biases introduced by remote data collection (e.g., variability in Zoom-recorded parent-child interactions, self-report biases). A brief discussion of mitigation strategies (e.g., standardized recording protocols, validation of online tools) is warranted.

13. While the authors mention imputation techniques, the protocol should specify which methods will be used (e.g., multiple imputation, sensitivity analyses) and how missing data assumptions will be tested.

14. The exclusion of non-English-speaking families due to language barriers is justified but raises equity concerns. The authors should elaborate on referral pathways for excluded families to ensure they receive comparable support.

15. The term "increased likelihood of NDD" (inclusion criteria) requires further clarification. For example, how are thresholds defined for developmental delay domains (e.g., "two or more domains")? The text alternates between "neurodevelopmental disability" and "neurodevelopmental disorder". Recommend consistent use of either "disability" or standardized terminology per DSM/ICD guidelines.

16. Suggest adding the table 'The schedule of enrollment', interventions, and assessments. The "Core Components of PACT Online" section in Table 1 could be optimized using bullet points for better readability.

17. Inconsistent reference formatting (e.g., journal name abbreviations, author initials) needs standardization per BMJ Open guidelines (e.g., using PubMed abbreviations).

VERSION 2 - AUTHOR RESPONSE

Response to Reviewer 1:

This study protocol presents a Hybrid Type 1 RCT evaluating the effectiveness and implementation of PACT Online, an online parenting intervention grounded in Acceptance and Commitment Therapy (ACT) for parents of children with neurodevelopmental disabilities (NDD). The manuscript addresses a critical gap in accessible, tailored support for this population. The integration of effectiveness and implementation analyses, alongside a robust consumer engagement strategy, strengthens the potential for real-world impact.

Response: Thank-you.

1. Why were families with children aged 0-10 years selected? What is the rationale for this age range?

Response: The age range of 0-10 years was chosen so that PACT Online could be available as early as possible, as soon as neurodevelopmental disability was suspected to

developmental delay identified. This has now been added to the paper under inclusion criteria and reads:

“The age range of 0-10 years was chosen so that PACT Online could be available as early as possible, as soon as neurodevelopmental disability was suspected to developmental delay identified.”

2. Which family members participated - fathers, mothers, or both? The description in the text is unclear.

Response: All parents and significant familial caregivers will be invited to participate; however, families will not be excluded if only one parent is able or willing to participate.

3. The inclusion criteria focus more on children. What are the inclusion conditions for parents?

Response: There are no inclusion conditions for parents. We have presented the full inclusion criteria in text.

4. Was the sample size calculation based on a pilot study? What software or formula was used for sample size calculation? The paper lacks a discussion section.

Response: Yes, the sample size calculation is based on a pilot study. The calculation used using Stata statistical software v14 (StataCorp, College Station, TX, USA). This is stated in text as follows:

> “Based on a previous RCT conducted by our team in 67 families of children with a neurodevelopmental disability (cerebral palsy) [25] we assume the standard deviation (SD) of the Global EAS will be 14.3 units, and that identifying a difference between the active (PACT) and waitlist control group of 5.0 units is clinically important. This is equivalent to a between-group difference of 0.35SD. To achieve 80% power, 258 families (129 in each group) are required to provide outcome data (alpha=0.05). To allow for expected 11-15% participant attrition (as experienced in our previous trials; [25] we will recruit a total of 300 families into this RCT. Participant retention strategies will be guided by the consumer engagement panel and will include a quarterly study update. Sample size calculations were conducted using Stata statistical software v14 (StataCorp, College Station, TX, USA).”

Protocol papers, including protocol papers published in BMJ Open do not always have a discussion section. It is not unusual for protocol papers to end with an ‘ethics and dissemination’ section as we have done. We prefer the current structure.

5. The description of the control group "Care-as-Usual (CAU)" is insufficient. Recommend adding detailed CAU descriptions (e.g., types of routine services, frequency) to clarify inter-group differences.

Response: CAU will vary from family to family and will consist of accessing medical and allied health interventions. We will collect information on which services each family accessed throughout the study. We have clarified this in text which now reads:

“Families randomized to the CAU (waitlist control) group will receive their usual services (e.g. medical, allied health, therapy) during the study participation period and will be offered the *PACT Online* intervention after completing the T3 follow-up assessment (at

6 months post baseline). We will collect information on which services each family accessed throughout the study."

6. Only outcome assessors were blinded, while participants and intervention implementers were not, which may introduce performance bias. This limitation should be explicitly addressed in the discussion.

Response: Protocol papers, including protocol papers published in BMJ Open do not always have a discussion section. It is not unusual for protocol papers to end with an 'ethics and dissemination' section as we have done. We prefer the current structure. We have clarified the bias potentially introduced in the Randomisation and Blinding section which now reads:

"Neither participants nor clinicians can be blind to the group allocation, and we acknowledge this may be a confound."

7. The plan to interview 30-40 parents lacks explanation of sampling strategy (e.g., maximum variation sampling) or data saturation assessment. Recommend adding justification for qualitative study design.

Response: We have made revisions to expand our explanation of the sampling strategy, and explicate how we will assess data saturation. We have also added further justification for the qualitative study design, aligned with the CFIR – Consolidated Framework for Implementation Research – framework. It now reads:

"To gain in-depth insight into factors shaping implementation acceptability, interviews were selected to support a Consolidated Framework for Implementation Research (CFIR) qualitative study design [29], prioritizing individuals' experiences of the intervention, settings and processes. Purposive sampling criteria (e.g., age of child, carer relationship) will be defined iteratively after the first 10 interviews; recruitment will conclude (n= 30-40) with saturation: when analysis produces no new themes. Grounded in CFIR [29], the interview guide prioritizes discussion of…"

8. While the Good Health Practices Scale (GHPS) was used for parental health behaviors, there's no mention of its psychometric validation in NDD parent populations. Psychometric properties (reliability/validity) need documentation. The low internal consistency (α=0.49) of the "affect quality" subscale in EA-SR may affect result reliability, requiring researchers' attention.

Response: We have clarified the GHPS psychometric validation so that it is now clear. It reads:

"Good Health Practices Scale (GHPS; 16 items) measures health behaviour in adults and has been validated in community samples, including by corelating with physiological biomarkers of poor health [44]."

Planned responses if there is low internal consistency on the affect quality subscale of the EA-SR are now documented and it reads:

"Good reliability estimates for the subscales have been found ranging from .71 and .84, with the exception of the affect quality subscale (α=.49) [28] which will be excluded from analysis if sufficient reliability is not achieved."

9. While the study aims to recruit Australia-wide, there is limited discussion of strategies to

ensure diversity (e.g., socioeconomic status, cultural/linguistic backgrounds). Given the exclusion of non-English-speaking families, the authors should address how this impacts generalizability and whether plans exist for future adaptations (e.g., translations).

Response: We have added clarity on strategies to ensure a representative sample as well as the impact of the exclusion of families that don't speak English on generalisability. It now reads:

"Australia-wide recruitment, recruitment through schools and deliberate recruitment from regional and rural areas via BUSHKids will maximise representativeness of the sample. It should be noted that generalisability will be limited to English-speaking families"

10. The use of the Consolidated Framework for Implementation Research (CFIR) is appropriate, but the manuscript lacks specificity about which CFIR domains/constructs will guide qualitative data collection and analysis. A brief table or paragraph mapping CFIR constructs to interview questions/analysis goals would enhance clarity.

Response: We have added detail for clarity which now reads:

The interview guide was developed, grounded in CFIR [29], with the purpose of understanding acceptability, appropriateness and feasibility of implementing the intervention with parents, as well as identifying contextual factors that act as facilitators and barriers to implementation success within the individual, inner setting and outer setting domains (see supplemental materials for interview guide).

In addition, the interview guide is attached as supplemental material.

11. When using Zoom to record parent-child interactive videos, it is necessary to explain the data storage method (such as encrypted servers), access control, and video destruction plan to comply with ethical requirements.

Response: The recordings will be stored on UQ servers with state-of-the-art data security meeting the requirements of the National Health and Medical Research Council and the Australian Code for Responsive Conduct of Research and accessible to study personnel only. Data will be destroyed after 15 years in accordance with UQ policy. This has been clarified in text and now reads:

"All assessments will be conducted remotely and/or online using REDCap for questionnaires and Zoom videoconferencing for collection of the EAS observational assessment footage and will be stored on REDCap and/or UQ servers with state-of-the-art data security meeting the requirements of the National Health and Medical Research Council and the Australian Code for Responsive Conduct of Research and accessible to study personnel only. Data will be destroyed after 15 years in accordance with UQ policy."

12. The manuscript does not address potential biases introduced by remote data collection (e.g., variability in Zoom-recorded parent-child interactions, self-report biases). A brief discussion of mitigation strategies (e.g., standardized recording protocols, validation of online tools) is warranted.

Response: The EAS is valid for naturalistic observations in home settings. Variability in the environment is not an issue. The remote recording does not introduce a bias for this tool. For clarity we have added:

“Extensive use of the EAS has demonstrated the excellent construct validity of this scale in establishing the expected relationship between EA and parent-child attachment including in naturalistic observations in home settings.”

Response: We have added an acknowledgement of the limitations of self-report measures which reads as following:

“The limitations of self-report measures are acknowledged.”

13. While the authors mention imputation techniques, the protocol should specify which methods will be used (e.g., multiple imputation, sensitivity analyses) and how missing data assumptions will be tested.

Response: We will conduct a sensitivity analysis and use multiple imputation by chained equations if the data is missing at random or pattern mixture models if the data is not missing at random. This is stated in the paper as follows:

“In the event that >10% of outcomes are missing, we will conduct a sensitivity analysis to investigate the sensitivity of results. The pattern of missingness will be examined, and if the data is ‘missing at random’ we will use multiple imputation by chained equations, whereas if the data is determined to be ‘missing not at random’ we will use pattern mixture models.”

14. The exclusion of non-English-speaking families due to language barriers is justified but raises equity concerns. The authors should elaborate on referral pathways for excluded families to ensure they receive comparable support.

Response: We have elaborated on referral pathways, and it now reads:

“Any families ineligible for from the study based on language will be referred to other services, for example parenting programs or allied health services, as appropriate.”

15. The term "increased likelihood of NDD" (inclusion criteria) requires further clarification. For example, how are thresholds defined for developmental delay domains (e.g., "two or more domains")? The text alternates between "neurodevelopmental disability" and "neurodevelopmental disorder". Recommend consistent use of either "disability" or standardized terminology per DSM/ICD guidelines.

Response: The thresholds for developmental delay domains are set to the definition used by the National Disability Insurance Scheme (NDIS) in Australia. We have added text to clarify this:

“…a diagnosis of developmental delay as defined by Australian National Disability Insurance Scheme (NDIS; i.e. substantial reduction in functional capacity) in two or more domains (i.e., gross motor, fine motor, language, or personal/social)…”

We have also replaced disorder with disability throughout.

16. Suggest adding the table 'The schedule of enrollment', interventions, and assessments. The "Core Components of PACT Online" section in Table 1 could be optimized using bullet points for better readability.

Response: The timing of interventions and assessments is already given clearly in the text. We think a table would be repetitive. We have ensure that Table 1 had bullet points for readability.

17. Inconsistent reference formatting (e.g., journal name abbreviations, author initials) needs standardization per BMJ Open guidelines (e.g., using PubMed abbreviations).

Response: Thanks. We have edited accordingly.
